# Trainable hardware for dynamical computing using error backpropagation through physical media

**DOI:** 10.1038/ncomms7729

**Published:** 2015-03-24

**Authors:** Michiel Hermans, Michaël Burm, Thomas Van Vaerenbergh, Joni Dambre, Peter Bienstman

**Affiliations:** 1 OPERA photonique, Université Libre de Bruxelles, Avenue F. Roosevelt 50, 1050 Brussels, Belgium; 2 ELIS Department, Ghent University, Sint Pietersnieuwstraat 41, 9000 Ghent, Belgium; 3 INTEC Department, Ghent University, Sint Pietersnieuwstraat 41, 9000 Ghent, Belgium

## Abstract

Neural networks are currently implemented on digital Von Neumann machines, which do not fully leverage their intrinsic parallelism. We demonstrate how to use a novel class of reconfigurable dynamical systems for analogue information processing, mitigating this problem. Our generic hardware platform for dynamic, analogue computing consists of a reciprocal linear dynamical system with nonlinear feedback. Thanks to reciprocity, a ubiquitous property of many physical phenomena like the propagation of light and sound, the error backpropagation—a crucial step for tuning such systems towards a specific task—can happen in hardware. This can potentially speed up the optimization process significantly, offering important benefits for the scalability of neuro-inspired hardware. In this paper, we show, using one experimentally validated and one conceptual example, that such systems may provide a straightforward mechanism for constructing highly scalable, fully dynamical analogue computers.

## Abstract (web summary)

Machine learning systems use algorithms that can interpret data to make improved decisions. Hermans *et al*. develop a physical scheme for a computing system based on recurrent neural networks that physically implements the error backpropagation algorithm, thus performing its own training process.

## Graphical abstract

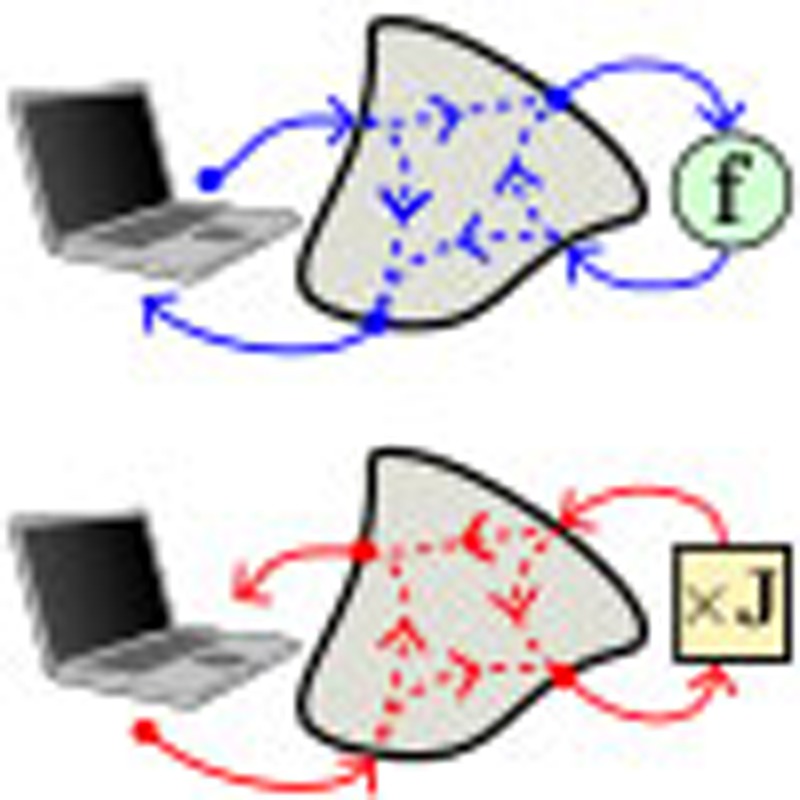

In a variety of forms, neural networks have seen an exponential rise in attention during the last decade. Neural networks trained with gradient descent are currently outperforming other, more classical approaches in a broad number of challenging tasks. One often-quoted result is their state-of-the-art performance in computer vision^1^, an example of a problem that is considered easy for humans, and hard for conventional computer algorithms. In combination with a number of semi-heuristic methods, this result has been obtained using the backpropagation algorithm, a method that has been around since the 1960s.

One highly interesting set of neural architectures are so-called recurrent neural networks (RNN), that is, neural networks that have temporal feedback loops. This makes them highly suited for problems that have a natural time element, such as speech recognition^2^ and character-based language modelling^3^,^4^. Conventional neural networks may be limited in solving such tasks, as they can only include a finite temporal context (usually a window of data of a fixed duration). RNNs, on the other hand, can—at least in principle—have indefinitely long memory. In addition, it is believed that temporal feedback loops are primary functional components of the human brain.

Currently, (recurrent) neural networks are most often implemented in software on digital devices, mostly for reasons of convenience and availability. At their core, however, these networks are analogue computers, and they come far closer to mimicking the workings of the brain than classic computation algorithms do. A computer made up of state-of-the-art components that matches the processing power of the human brain is estimated to consume about 0.5 GW of power^5^, a full seven orders of magnitude more than the 20 W required by the brain itself. If we ever wish to achieve any degree of scalability for devices performing brain-like computation, we will need to embrace physical realizations of analogue computers, where the information is encoded in hardware by continuous physical variables, and where these data are processed by letting them interact through physical nonlinear dynamical systems.

One line of research that has been partially successful in accomplishing this goal is that of reservoir computing (RC)^6^. This paradigm, which combines several previous lines of research^7^,^8^, essentially employs randomly constructed dynamical systems to process a time-varying signal. If the system is sufficiently varied, nonlinear and high dimensional, it acts as an efficient random feature generator, which expands the input signal into a high-dimensional space in which the time series-processing problem becomes much easier to solve. All that remains to be optimized is a a very small number of meta-parameters, and a linear mapping of these features to a desired output signal, which can be performed efficiently with any linear algebra solver.

The last decade of research into RC has shown a variety of interesting examples of physical implementations of analogue processors. It has been shown to work with water ripples^9^, mechanical constructs^10^,^11^, electro-optical devices^12^,^13^, fully optical devices^14^ and nanophotonic circuits^15^,^16^. Despite some remarkable successes, the RC concept still faces the problem of being ineffective for tasks that require a great deal of modelling power (for example, natural language processing, video stream processing and so on). The main reason is that any particular feature that needs to be extracted from the input data has to be present in the randomly constructed nonlinear feature space offered by the reservoir. When the input dimensionality grows, the probability of having the correct features present becomes extremely small. Even for moderately demanding tasks, this means that good performance requires a disproportionally large dimensionality of the system at hand, up to tens of thousands of state variables in practice^17^. By contrast, neural networks trained by the backpropagation algorithm can build the required nonlinear features internally during the training process, which makes them far more scalable.

In recent work, we have shown that it is possible to extend backpropagation (in particular the variant used to train RNNs, called backpropagation through time (BPTT)^18^) to models of physical dynamical systems^19^. We have shown that it can serve as an efficient automated tool to find highly non-trivial solutions for complex dynamical problems. In ref. 20, we have shown that backpropagation can be used on models of existing electro-optical reservoirs, offering a method to optimize their input encoding. So far, this work relies on simulation, however. The backpropagation algorithm operates on a computer model of the dynamical system. If we would use it to train physical analogue computers, we would face the same scaling issues that we encountered with systems that mimic the human brain.

In the following, we offer a definition of a set of specific physical dynamical systems that can act as analogue computing systems. Crucially, we show that—in contrast to the results presented in ref. 19—the backpropagation algorithm can be performed physically on such systems, with only minor additional hardware requirements and without the need for simulation. This approach significantly reduces the external computational demands of the optimization process, hence greatly speeding it up. First, this means that we can overcome the aforementioned limitations of the RC paradigm by building systems that are more optimized for a specific task. Second, it implies that we can break barriers in terms of scalability: an analogue physical computing set-up is inherently massively parallel, and its speed of operation does not directly depend on its complexity. Whereas training systems to perform complex, large-scale machine learning tasks currently take several days to complete, physical hardware as introduced in this paper may offer a way to reduce this to more manageable times.

## Results

### Theory

In this section, we will describe the general physical system we use to implement machine learning models, and we explain how their reciprocity allows to perform the training process in hardware. We start by introducing an abbreviated notation for a multivariate convolution. If **x**(*t*) is a multivariate signal and **W**(*t*) a matrix with time-varying elements defined for *t*>0, we define the signal **y**(*t*) as the convolution of **x**(*t*) with **W**(*t*) as follows:


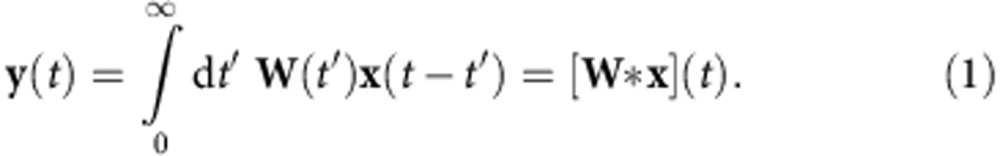


We will consider systems as follows. We will assume that there are a set of *N* signal input sources *s*_*i*_(*t*) that excite the linear dynamical system (LDS) and a set of *M* output receivers that receive an output signal *o*_*i*_(*t*). We can write both the sets as a single source and receiver vector **s**(*t*) and **o**(*t*), respectively. The LDS will cause the following transformation between the source and receiver:


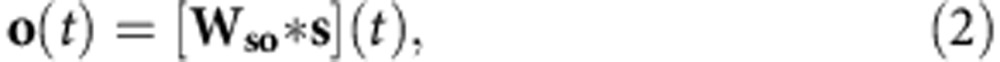


where the impulse response, or first-order Volterra kernel **W**_**so**_(*t*) characterizes the transfer function of the system. Furthermore, we are able to use the LDS reversely, where the receivers now act as sources and the sources act as receivers. In this case, **s′**(*t*) acts as the input of the system and **o**′(*t*) represent the received signal at the places of the original sources. If the LDS is a reciprocal system, the following equation holds:


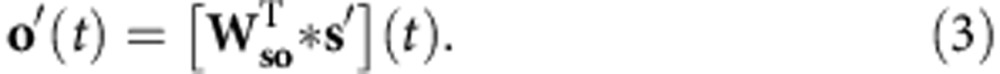


This property is crucial, as it will allow us to perform error backpropagation (which we introduce later on) physically. Reciprocal systems are ubiquitous in physics. One important example of such a system, which we will use for the rest of the paper, is the propagation of waves through a linear medium. Suppose for instance we have a chamber in which we place a set of different speakers and a set of microphones, the signals the microphones receive would indeed be described by equation (2), where **W**_**so**_(*t*) would be determined by the shape of the room, the absorption of the walls, the air density and so on. If we then replace each speaker by a microphone and vice versa, the signal we received would be described by equation (3). Another set of examples are systems described by the linear heat (or diffusion) equation. Here the source would have a controllable temperature, and the receivers would be thermometers, where the medium performs an operation as described by the above equations.

**Figure 1:**
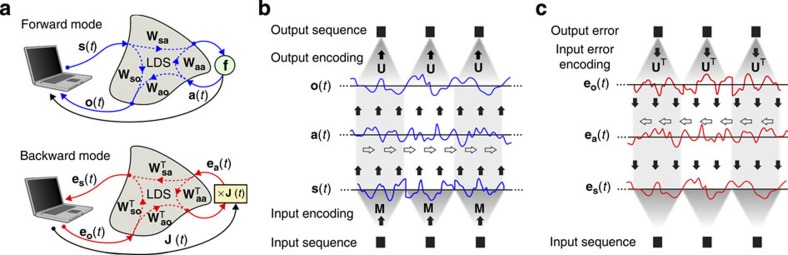
Schematic of physically implemented backpropagation. (**a**) Illustration of the most general set-up of the physical neural network studied in this paper. The top diagram shows how the signals propagate through the system and the nonlinear feedback with blue arrows during the forward pass. The filter operations are depicted with dashed lines running through the LDS, which is depicted as a grey blob. The bottom diagram shows the error backpropagation phase, where the signal runs backwards through all functional dependencies. Here filter operations run in the opposite direction such that they are represented by the transpose of their impulse response matrices. Note that the computer is not in the loop during the forward or backward pass, but only serves to send out a predefined signal, and to record at the same time. (**b**) Depiction of the masking principle in the forward direction. At the bottom, we see three consecutive instances of an input time series. Each of these is converted into a finite time segment through the masking signals **M**(*t*). These segments are next concatenated in time and serve as the input signal **s**(*t*) for the dynamical system (where time runs according to the white arrows), and which in turn generates an output signal **o**(*t*). The output signal **o**(*t*) is divided into finite length pieces, which are decoded into output instances of an output time series using the output masks **U**(*t*). (**c**) The backpropagation process happens in a completely similar manner as in the forward direction. This time, the transpose of the output masks serve as the encoding masks. Finally, the input error signal **e**_**s**_(*t*) is also segmented in time before it is used to determine the gradients w.r.t. **M**(*t*).

Linear systems can only perform linear operations on their input signal. In order for the full system to be able to model nonlinear relationships, we add nonlinear feedback. We provide a set of *M*_**a**_ additional receivers and *N*_**a**_ sources. The signal that is detected at these new receivers gets sent through a nonlinear operator 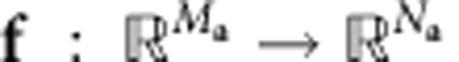 and is fed back into the system via the new sources. We denote the signal after the function as **a**(*t*). The impulse response matrix for the transition from the input sources to the receivers for the nonlinear feedback we denote as **W**_**sa**_(*t*), and those for the transition from the nonlinear feedback sources to the output receivers and nonlinear feedback receivers with **W**_**ao**_(*t*) and **W**_**aa**_(*t*), respectively. A schematic diagram of the full system is shown in the top of Fig. 1a. The system is described as follows:


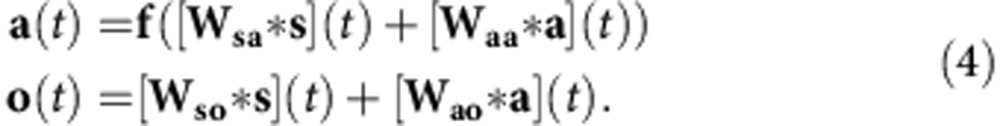


As we argue in Supplementary Note 1, for specific choices of the impulse response matrices, these equations reduce to those of neural networks, including all kinds of deep networks, RNNs and so on. Neural networks are usually trained using gradient descent based on backpropagation. Therefore, if we wish to use this system as a trainable model for signal processing, we need to be able to calculate gradients for the parameters we can change (the impulse response matrices and the input and output encoding which we define later). First of all, we define a cost functional *C*(**o**(*t*)), a functional of the whole history of **a**(*t*) in the interval *t*ε{0⋯*T*} that we wish to minimize. This could, for instance, be the time integral of the squared difference between the actual and a desired output signal. Next we define the partial derivative of *C*(**o**(*t*)) with respect to (w.r.t.) **o**(*t*) as **e**_**o**_(*t*). The error backpropagation process is then described by the following equations:


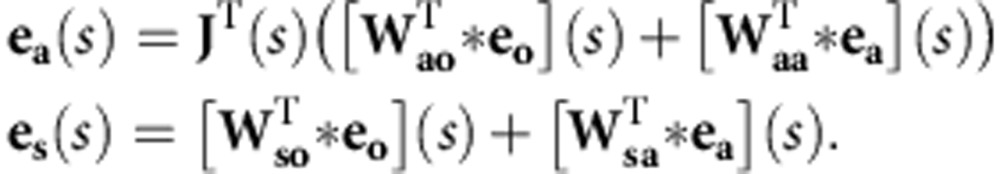


Here *s*=*T*−*t*, that is, the equations run backwards in time and **J**(*t*) is the Jacobian of **f** w.r.t. its argument. From the variables **e**_**a**_(*t*) and **e**_**s**_(*t*), gradients w.r.t. all impulse response matrices within the system, and w.r.t. **s**(*t*) can be found, which in turn can be used for optimization (see Supplementary Note 2 and Supplementary Fig. 1 for the derivations and for the mathematical interpretation of **e**_**a**_(*t*) and **e**_**s**_(*t*)). The crucial property of these equations is that, thanks to the reciprocity of the LDS, they too can be performed physically on the same system. First of all, we consider the time *s* as physical time running forwards (which in practice means that we need to time-reverse the external input signals **e**_**o**_(*t*) and **J**(*t*)). If we then switch the positions of the sources and receivers (leading to transposed impulse response matrices), and instead of providing nonlinear feedback, we modulate the feedback with 
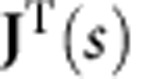
, we have physically implemented equation (5), and hence can record **e**_**a**_(*s*) and **e**_**s**_(*s*) directly from the system. This principle is depicted in the bottom of Fig. 1a. In summary, the requirements for our system are a reciprocal LDS, a physical implementation of the nonlinear feedback and a physical implementation of time-varying modulation with **J**(*s*).

In many cases, it is difficult to send signals to multiple sources and to record signals from multiple receivers due to the cost and complexity associated with the necessary hardware. Yet a high-dimensional state space is desirable as it increases the complexity and therefore the representational power of the system. To increase the effective dimensionality of the system, we use an input–output multiplexing scheme that was first introduced in ref. 21. This increases the effective dimensionality of the system and its parameters can also be optimized using the physical backpropagation method. Suppose we have an input time series **x**_*i*_ that we wish to map onto an output time series **y**_*i*_. First of all, we define an encoding that transforms the vector **x**_*i*_, the *i*-th instance of the input data sequence, into a continuous time signal segment **s**_*i*_(*t*):


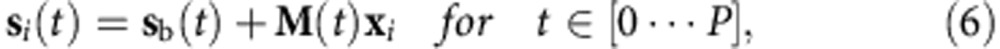


where *P* is the masking period and **s**_b_(*t*) is a bias time trace. The matrix **M**(*t*) contains the so-called input masks, defined for a finite time interval of duration *P*. The input signal **s**(*t*) is now simply the time concatenation of the finite time segments **s**_*i*_(*t*).

The output decoding works in a very similar fashion. If there is a time series **y**_*i*_ that represents the output associated with the *i*-th instance of the input time series, we can define an output mask **U**(*t*). We divide the time trace of the system output **o**(*t*) into segments **o**_*i*_(*t*) of duration *P*. The *i*-th network output instance is then defined as


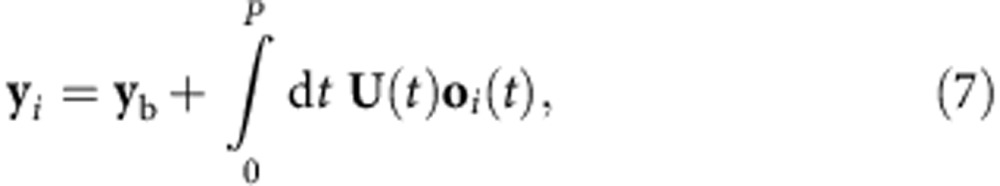


with **y**_b_ a bias vector. Effectively, the whole time trace of **o**_*i*_(*t*) now serves as the immediate state vector of the system, drastically increasing its effective dimensionality.

The process described here is essentially a form of time multiplexing, and is depicted in Fig. 1b. The backpropagation phase happens in a similar fashion. Suppose we have a time series with instances **e**_*i*_, which are the gradient of the chosen cost function w.r.t. the output instances **y**_*i*_. Completely equivalent to the input masking, we can now define the error signal **e**_**o**_(*t*) as a time concatenation of finite time segments 
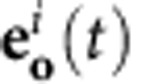
:


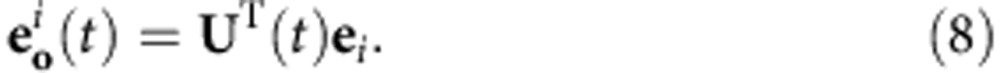


Using this signal as an input to the system during backpropagation will provide us with an error **e**_**s**_(*t*), which in turn can be used to determine the gradient for the masking signals **M**(*t*) as depicted in Fig. 1c (for the derivations, see Supplementary Note 3). Note that the signal encoding and decoding happens on an external computer as well, and not physically. These operations can be fully parallelized, however, such that we do not lose the advantages of the processing power of the system. In ref. 20, we showed in simulation that optimizing input masks using BPTT on a computer model of an electro-optical set-up as used in ref. 12 had significant benefits. As we will show experimentally, physical backpropagation allows us to largely omit the modelling and simulation, and allow for a more direct optimization.

The full training process works as follows. First, we sample a representative amount of data, encode it to a continuous time input signal, run it through the physical system and record the output signals. Next, we compute the cost corresponding to the output and construct the signal **e**_**o**_(*t*) (on the computer), which we then also run through the system physically, and record the signal **e**_**s**_(*t*) (and **e**_**a**_(*t*) if applicable). Once this is completed, we can compute gradients w.r.t. the relevant parameters on the computer. We then subtract these gradients from the parameters, multiplied with a (small) learning rate. This process is repeated for many iterations until we reach satisfactory performance. All the operations that take place on the computer can be performed fully in parallel, and hence do not significantly slow down the training process.

### A real-life acoustic set-up

We have tested the principles described above by building a system that uses the propagation of acoustic waves as an LDS. To reduce the complexity of the set-up, we work with only one signal source (a small computer speaker) and one receiver (a voice microphone). The sound enters a 6-m long plastic tube via a paper funnel, and the microphone receives the signal at the other end of the tube. The tube delays the signal and introduces reflections and resonance frequencies. Note that, due to the fact that the signal in this case is scalar, all impulse response matrices determining the system are scalar too, which means that they are equal to their transpose. This has the advantage that we do not need to switch the speaker and microphone between the forward and backward phase.

The received signal is electronically truncated at 0 V (such that only positive voltages can pass), implementing a so-called linear rectifier function, which acts as the system nonlinearity **f**. The linear rectifier function is currently a popular activation function in neural architectures^22^. Feedback is implemented by adding this signal to the external input signal. One important advantage of the linear rectifier function is that its derivative is a binary signal: equal to one when the signal is transmitted, and equal to zero when it is cutoff. This means that multiplication with the Jacobian is equivalent to either transmitting the feedback signal unchanged, or setting it to zero, which can be easily implemented using an analogue switch. For a more detailed explanation of the relation between the acoustic system and the general case described by equation (4), we refer to the methods section. Note that other types of nonlinearity such as, for example, a sigmoid and its corresponding derivative, can also be implemented in analogue hardware (see for instance ref. 23).

We have used the physical backpropagation set-up to train input and output masks. Note that in principle it could also be used to optimize properties of the acoustic set-up itself, but we have omitted this for reasons of experimental simplicity. We have tested the set-up on an academic task that combines the need for nonlinearity and memory. The input time series *q*_*i*_ is scalar and consists of a series of i.i.d. integers from the set {0,1,2}, which are encoded into an acoustic signal as described above. The desired output time series *y*_*i*_ is defined as


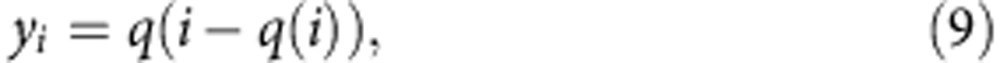


that is, the task consists of retrieving the input with a delay that depends on the current input. The fact that the delay is variable makes that the task is nonlinear, that is, it cannot be solved by any linear filtering operation on the input.

**Figure 2:**
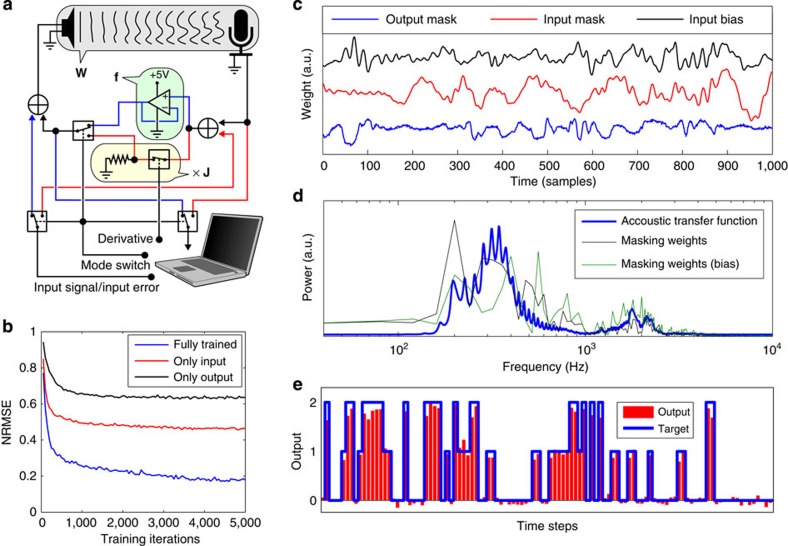
Results of the acoustic backpropagation experiment. (**a**) Schematic depiction of the electric circuit of the experiment. The grey box at the top represents the speaker, tube and microphone. The blue circuit lines are only in use during the forward propagation and the red lines are in use during the backpropagation phase. The forward or backward mode can be toggled by a logic signal that comes from the PC, and which controls three analogue switches in the circuit. In the forward mode, the nonlinear feedback is implemented by an op-amp voltage follower which cuts off the signal at 0 V (green box), implementing a linear rectifier function. In the backprop mode, the multiplication with the Jacobian is implemented by a fast analogue switch that either outputs zero, or transmits the signal. (**b**) Example of the normalized root mean square error (NRMSE) as a function of the number of training iterations for three cases. That where both the output and input masks are trained, and those where only one of each is trained. (**c**) Example of resulting input, bias and output masks after training, corresponding to **M**(*t*), **s**_b_(*t*) (equation (6)) and **U**(*t*) (equation (7)), respectively. (**d**) Example power spectrums of the input masks and the power spectrum of the transmission of the speaker–tube–microphone system. (**e**) Example of the network output versus the target.

For details concerning the experiments, we refer to the Methods section. A schematic depiction of the set-up and the main results of the experiments are shown in Fig. 2. In Fig. 2e, we show a comparison between the system output and the target, indicating that the system has learned to solve the task successfully. We show the evolution of the normalized root mean square error (NRMSE) during the training process in Fig. 2b. To make sure that the physical backpropagation works as intended, we have run two additional tests in which we trained either only the output masks (the RC approach, which does not require backpropagation) or only the input masks, keeping the other random and fixed. As can be seen in Fig. 2b, training only output or input masks in both cases reduces performance. Note that if the input masks are trained while the output masks are kept fixed and random, all adaptations to the system parameters are exclusively due to the error signal that has been propagated through the system in the form of sound. On top of this, the input mask training needs to find a solution that works with a completely random instantiation of the output mask. The fact that it can achieve this at least to some degree (NRMSE ≈ 0.47) demonstrates that physical BPTT works as intended.

Note that the result for fixed input masks and trained output masks is not meant to be interpreted as a fair comparison with the RC method, but rather as a naive baseline. Indeed, it is likely that the results for the untrained input masks could be improved by, for example, constructing the random input mask such that it has a spectrum matching the transmission of the acoustic system.

The input and output masks after training are shown in Fig. 2c, showing their temporal structure. In Fig. 2d, we show the power spectra of the input masks (and consequently the power spectra of the signals sent into the system) compared with the power spectrum of the system transmission (measured as the squared ratio in amplitude between the output voltage in the microphone and the input voltage in the speaker). Clearly, the acoustic parts of the full system (speaker–tube–microphone) only transmit certain frequency bands (a.o. the resonance frequencies of the tube are visible as a set of peaks.) and the input masks seem to have learned to match this spectrum. Note that at no point during training or testing we ever required a model of the acoustic part of the system. The impulse response matrices of the system do not need to be known for the backpropagation to work. Also important to mention is that no process on the external computer or in the electric circuitry provides the required memory for the system to process the time series. Instead, this happens due to the physical memory inherent to the acoustic system and to the nonlinear feedback, which the training process learns to exploit. This means that at any point in time, information about past inputs exists solely as acoustic waves travelling through the tube.

### A conceptual electro-optical implementation

The described acoustic implementation can be extended to a larger and faster system relatively easily. For instance, one could use ultrasound for higher data transmission (combined with a medium with a high speed of sound). Here one could use piezoelectric transducers that can both emit and receive signals. Sound propagation is in that sense an interesting platform for a physical analogue neural network. The most attractive medium, however, but also more technologically challenging, would be light. Light can transport information at a very high speed, and unlike sound waves, it can be easily guided through fibre optics and integrated photonic waveguides. Just like sound waves, light transmission is reciprocal, making it possible to perform error backpropagation physically on the system. Indeed, using light as a medium for neuro-inspired processing has been studied extensively in the past^24^,^25^. These examples primarily exploit parallel processing that happens when light travels through a (often holographic) medium. In our case, we would like to exploit not only parallelism, but also the time delays that are inherent to travelling light.

As a proof of concept, we propose a circuit to perform physical backpropagation electro-optically, partially inspired by the systems described in refs 12, 13. Delays are physically implemented by means of long optical fibres. For this example, we wish not just to train the input masks, butto control the way in which the signals are mixed as well. Concretely, if we have an *N*-dimensional state **a**(*t*), we wish to optically implement a mixing matrix **W** of size *N* × *N*, such that the mixing matrix **W**_**aa**_(*t*)=*δ*(*t*−*D*)**W**, where *D* is the delay introduced by the optical fibres. Set-ups for computing matrix–vector products optically have been experimentally demonstrated in the past^26^,^27^, and here we will assume that it is possible to perform them all-optically (see methods). Note that we do not need very high parameter precision for neural network applications, as the detection of the signal will be inherently noisy.

**Figure 3:**
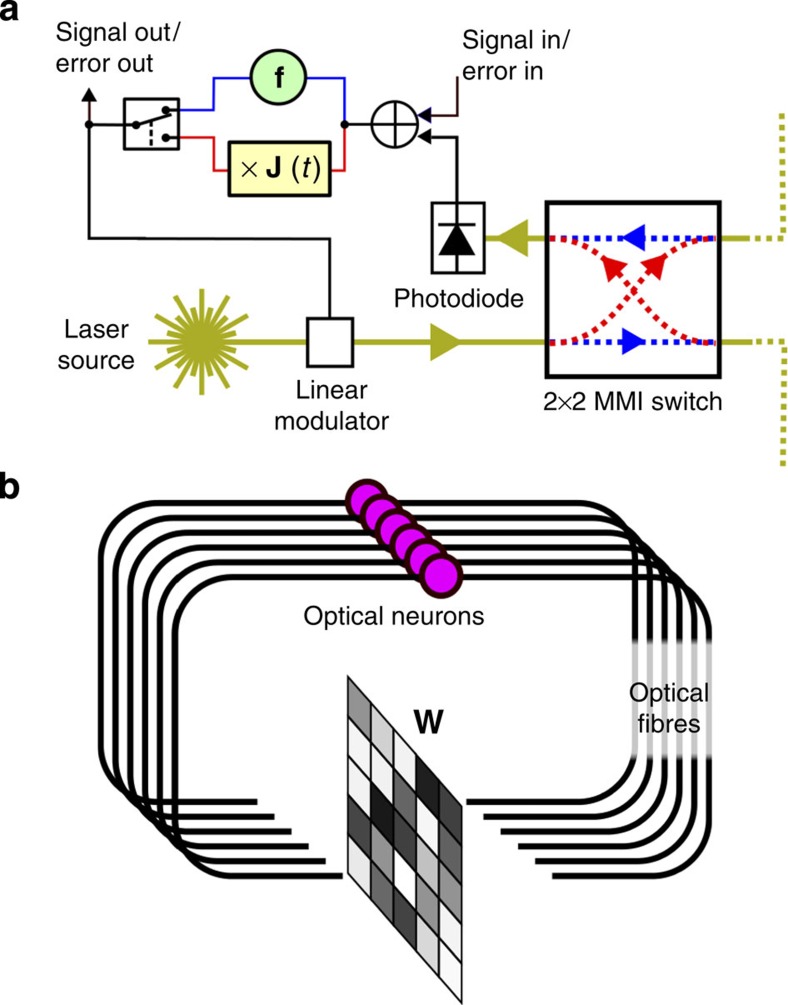
The electro-optical set-up. (**a**) Schematic depiction of an electro-optical neuron, where pathways that are exclusive in the forward or backward mode are depicted in blue or red, respectively. It is largely similar to the electronic circuit in the acoustic set-up, with the difference that the input now enters the system before the nonlinearity. The 2 × 2 switch allows light to travel either forwards or backwards through the fibre network (fibres depicted by yellow lines). (**b**) Depiction of a network of electro-optical neurons, each purple circle represents a neuron. They send their output signals through optical fibres (which also incorporate delay) to an optical matrix–vector multiplier which multiplies with a matrix **W** in the forward direction and **W**^T^ in the backwards direction. The elements of **W** can be set electronically, and are adapted in each training iteration. Note that each neuron also has an incoming and outgoing connection to external hardware that sends input and records output (not depicted).

Similar to the acoustic example, we conceive an electro-optical ‘neuron’ (see Fig. 3a), which sends and receives optical signals, and either applies a nonlinear function or multiplies with the Jacobian. We encode the state **a**(*t*) as light intensity. Each neuron will have a fixed-power laser source, which can be modulated between the minimal and maximal value. The nonlinear function can be simply implemented by electronically truncating the feedback signal in a range corresponding to a minimum and maximum intensity levels of the laser, conveniently making full use of the signal range. Note that such a behaviour can be implemented relatively easily in high speed electronics, as it is an inherent property of amplifiers used in optical telecommunication^28^. The Jacobian of such a function is again a simple binary function. Finally, we can send the light into the optical circuit either in the forward or backward direction by using a 2 × 2 optical switch.

In the final set-up, we simulate 20 electro-optical nodes. We add different levels of noise to the measured intensity. We applied the simulated version of this system on a realistic phoneme recognition task, which is part of speech processing and hence a typical example of a problem that can be solved using RNNs. We used the often-used TIMIT data set, which is a speech corpus in which the phonemes are directly labelled on the speech signal itself. As an error measure, we use the frame error rate, which is the fraction of frames (time steps) in the input signal that have been mislabeled. More details on the task can be found in the Methods section.

We ran a number of simulations that range from ideally (mathematically correct) implemented backpropagation to simulations which include unavoidable non-ideal behaviour for a real physical set-up. In particular, we included nonlinearity in the backpropagation phase, as well as measurement noise and the case in which the modulation with the Jacobian is only an approximation of the true Jacobian (see Supplementary Fig. 2). Details and the results of these experiments can be found in Supplementary Note 4 and Supplementary Table 1, respectively, as well as a discussion on the expected speed of the conceptual electro-optical set-up. What we found was a remarkable robustness against non-ideality for the task at hand, only increasing the test error to a limited degree compared with the ideal scenario. We obtain a test frame error rate of ~30%. Comparing this with the other results in literature, we find that we perform in the same ballpark as some other established machine learning techniques (see for instance an overview of results on frame error rate in ref. 29, where the results range from 25 to 39%). When we compare this with the RC paradigm, similar results are only obtained using extremely large reservoirs, with up to 20,000 nodes^30^.

## Discussion

In this paper, we have proposed a framework for using reciprocal physical dynamical systems with nonlinear feedback as analogue RNNs. We have demonstrated that the error backpropagation algorithm, which efficiently optimizes an RNN, can be implemented physically on the same system, thus greatly reducing the necessary computations required for the optimization process. This in turn paves the way to faster, more scalable analogue computing. We have experimentally verified the proposed system using a real-world acoustic set-up as a proof of concept, as well as a simulated electro-optical set-up. In this second, more complex set-up, we explored the impact of expected sources of non-ideal behaviour on the performance of a real-world task and where we demonstrated that good performance does not require a very precise physical implementation of the backpropagation algorithm, and can tolerate reasonable levels of noise and nonlinearity. We have also included a short discussion on what processing speed may be obtained for an electro-optical set-up in the Supplementary Discussion.

The concepts presented in this paper provide a sizeable step forward towards a novel form of processing, which relies far less on software implementations and comes much closer to brain-like computation, especially in the sense that the system can partially internalize its own training procedure.

By using analogue physical processes to compute, we may benefit from inherent massively parallel computing, great potential speed benefits and low-power usage, the properties that were the initial motivation for research into physical RC systems. Specifically, the obtainable speed does not depend on the dimensionality (number of sources and receivers) of the system, offering inherent scalability. The possibility to fully optimize all internal system parameters using the backpropagation algorithm offers great performance improvements, and makes the application domain of the proposed set of systems far greater (as has been evidenced in ref. 20). This also means that such physical set-ups can potentially become competitive with digitally implemented neural networks, which are currently the state-of-the-art for several important signal processing problems. The training process too benefits from being implemented physically, meaning that there is only limited need for external processing. If the physical system under consideration has speed benefits compared with a digitally implemented neural architecture, these benefits are also present for training the system. Finally, while physically implementing the training process comes at an additional complexity cost (modulation of the feedback with the jacobian), the benefits over optimization of the parameters in simulation is paramount. Optimization in simulation might require a very precise model, and it is hard to predict how model-reality discrepancies would manifest themselves once the parameters obtained in simulation are applied to the physical set-up. When instead optimizing on the physical system there can be imperfections like the ones we explored in Supplementary Note 4 but one is certain that the measurements used to base the training on are those from the real system, and no additional system characterization is needed to perform the training.

Important challenges for the large-scale execution of this scheme still remain. The current set-up still requires some level of external processing for computing the gradients. If the analogue part of the system is sufficiently fast, gradient computations may become the bottleneck of training, though this may be partially redeemed by the fact that they are solely matrix–matrix multiplications (without a sequential part), which means that it is fully parallelizable.

Recording the signals in the forward and backward pass still requires digitization. Currently, this is the most important hurdle to scaling up the system in practice, as analogue-digital conversion at high speeds is expensive and consumes a lot of power. This limits the number of sources and receivers that can be practically applied. Time multiplexing of the input, as used in both examples of this paper, partially solves this problem, but at the cost of reducing the obtainable speed of the system. Larger numbers of sources and receivers would also benefit more from the spatial parallelism that is offered by acoustic or optic systems.

With currently available hardware, one could potentially build physical systems that are competitive with digitally implemented neural networks, as we demonstrated using a simulated electro-optical set-up. Truly exploiting the full potential of analogue physical computation, however, very likely requires the design of novel hardware that internalizes all necessary elements into a single device. In particular, future research into this topic should explore ways to develop hardware which has impulse responses determined by large amounts of controllable parameters. This would increase the number of trainable parameters and hence the representational power of the systems. Finally, it is also of importance to relate the results found in this paper to developments in neural network research. For one, it was recently found^31^ that random feedback weights for the backpropagation phase can also be used to train feedforward networks. It should be investigated if this has implications for the recurrent systems under considerations in this paper.

## Methods

### Acoustic experiments

For the acoustic set-up, we used a data acquisition card, which samples the signal at 40 kHz (well above the maximum frequency that is still passed through speaker–tube–microphone system). Input and output masks consist of 1,000 samples, which means that time series are processed at a rate of 40 time steps s^−1^.

We train the input and output masks over the course of 5,000 iterations. Initial values for the input and output masks are picked by independent, identically distributed sampling from a normal distribution with zero mean and variance equal to 0.2 and 0.1, respectively. Each training batch consists of a newly generated time series of 100 instances. This means that each training iteration (forward and backward pass) takes ~5 s, and the complete training takes about 7 h. We found little to no significant variation in performance for different random initializations of the input and output masks.

As absolute scaling of the error signal does not matter for the backpropagation phase, we always normalize and rescale the error signal before we use it as the input. This ensures that the signal always remains well above the noise levels. Note that this step causes us to lose the absolute magnitude of the gradients. For parameter updates, we therefore normalize the obtained gradients before using them. The learning rate is set to 0.25 at the start of the experiment, and then linearly decays to zero to ensure convergence at the end.

Mathematically, the full system can be described as follows: let *s*(*t*) be the input signal after the encoding and *a*(*t*) the (scalar) state of the system at time *t*, then


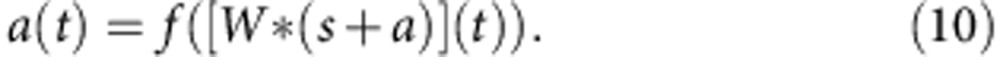


Here *f*(*x*) = *max*(0,*x*) is the linear rectifier function and **W**(*t*) is the scalar impulse response of the speaker–tube–microphone system, that is, it is the signal that would be received by the microphone for a Dirac delta voltage impulse for the speaker. The output of the system is also the state *a*(*t*). This means that we can relate the acoustic system to the general case of equation (4) if **W**_**sa**_(*t*)=**W**_**aa**_(*t*)=*W*(*t*), **W**_**ao**_(*t*)=*δ*(*t*) (the Dirac delta function) and **W**_**so**_(*t*)=0.

### Conceptual photonic set-up

The system is described by the following equation:


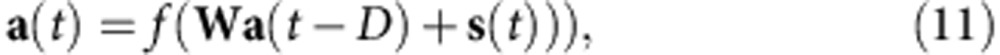


where **W** is the matrix, which is implemented optically (see below). The function *f*(*x*) truncates the signal between minimal and maximal intensity (which we define as −1 and 1, respectively):


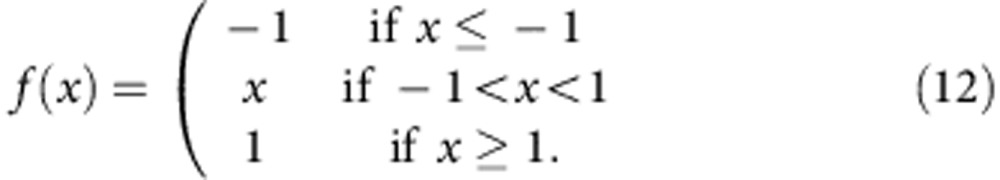


For what follows, we assume that all the nodes’ laser sources have different wavelengths, such that light intensities add up linearly. Note that this is feasible within the presented set-up: optical 2 × 2 switches can have bandwidths that exceed 100 nm (ref. 32, whereas laser bandwidths are usually of the order of 1 nm or less. If we assume laser light with a wavelength of ≈ 1 μm, and we assume that the different wavelengths differ as little as 1 nm between the nodes, the slowest resulting fluctuations in intensity from adding up these signals are of the order of 100 GHz (derived form the difference in their frequencies), 100 times faster than the assumed speed at which we measure the signal. An alternative approach would be to use light sources that are inherently broadband (for example, not lasers but light-emitting diodes, which have a bandwidth of 50–100 nm).

Several ways to implement optical matrix–vector multiplication have been discussed in literature. One possible method would be to encode the vector **a** as light intensities. We can let the light pass through a spatial array of tuneable intensity modulators and focus the light on the other side to perform a matrix–vector product. Conversely, if the light comes from the other direction, the signal is effectively multiplied with the transpose of the matrix. Directly implementing the whole concept using intensities to encode the signal would have the important limitation that all elements of **W** would be positive, as light intensities can only be added up. This is a strong drawback, as this would mean that the system can only provide weighted averages of the individual states and cannot use and enhance differences between them. Therefore, we envision a different approach where each element of **a** is encoded by two light signals with intensities: **k**+**a** and **k**−**a**, with **k** a vector with all elements equal to one. This ensures that the intensities fall into a positive range between 0 and 2, which can correspond to the minimum and maximum output intensity level of each neuron. Now, these constituents are sent to two separate arrays of intensity modulators **W**_1_=**K**+**W**/2 and **W**_2_=**K**−**W**/2, where **K** has all elements equal to one. As all elements of **W**_1_ and **W**_2_ need to be positive, this means that the range of the elements of **W** fall within the range −2 to 2. If we combine the light signals after the intensity modulation and hence add up the intensities we get:


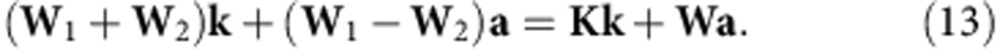


The first term will simply introduce a constant bias value, which we can remove electronically after measuring. The second term now contains the matrix–vector product where the elements of **W** can be both positive and negative.

For the simulations, we use a piecewise constant input signal with a fixed sample period (equal to the sample period of the measurement). We again used the masking scheme, where each masking period consisted of 50 sample periods. We chose the delay *D* at 51 sample periods and used a network of 20 optical neurons.

The TIMIT data set^33^ consists of labelled speech. It has a well-defined training and test set, which makes comparison with results in literature possible. Each time step (frame) needs to be labelled with one out of 39 possible phonemes. The input signal consists of a 39-dimensional time series (the fact that it has the same dimensionality as the output signal is coincidental), which encodes the speech signal using MFCCs. For more details on how the data is encoded, please check, for example, ref. 30.

We trained for 50,000 iterations. In each iteration, we randomly sampled 200 sequences of 50 frames from the training set. We again normalized the gradients. Parameter updates were performed with a learning rate starting at 1, which linearly dropped to zero over the course of the training.

## Author contributions

M.H. conceived, designed and conducted the physical and simulated experiments, M.B. contributed in building the electronic hardware for the acoustic experiment, T.V.V. co-designed the simulated experiments and helped conceive the electro-optical concept. J.D. and P.B. supervised the experiments and the project.

## Additional information

**How to cite this article:** Hermans, M. *et al*. Trainable hardware for dynamical computing using error backpropagation through physical media. *Nat. Commun*. 6:6729 doi: 10.1038/ncomms7729 (2015).

## Supplementary Material

Supplementary Information Supplementary Figures 1-2, Supplementary Table 1, Supplementary Notes 1-4, Supplementary Discussion and Supplementary References
