# Supplementary material for: Trainable hardware for dynamical computing using error backpropagation through physical media
Source: Nat Commun. 2015 Mar 24;6:6729. doi: 10.1038/ncomms7729 (PMC4382991; doi:10.1038/ncomms7729)
Supplement: Supplementary Information — Supplementary Figures 1-2, Supplementary Table 1, Supplementary Notes 1-4, Supplementary Discussion and Supplementary References [file ncomms7729-s1.pdf]

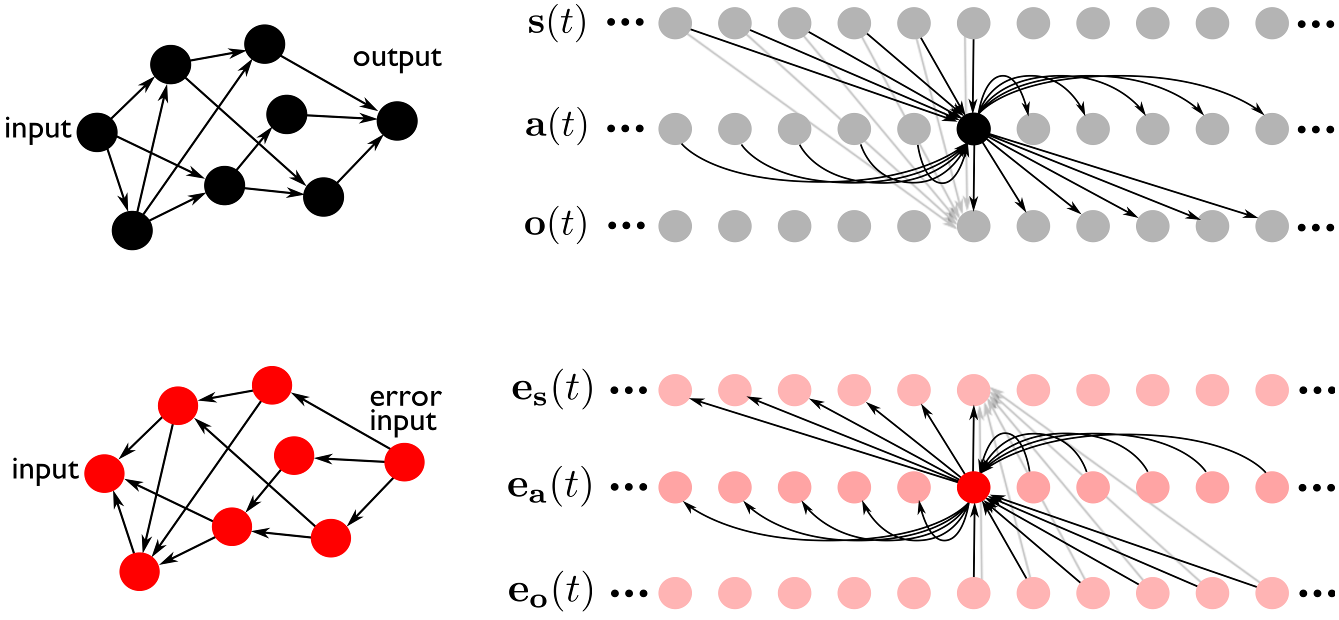

Supplementary Figure 1: Illustration of the graph representation of a neural network architecture. On the left we have depicted the forward and backward graph. Each black circle represents a summation and (optionally) a nonlinear function (except for instance at the output and input nodes). Each arrow represents a linear transformation matrix. During the error backpropagation phase, the direction of all the edges is reversed, and the error is propagated backwards through the network. Once each node is associated with an error, the transition matrices can be updated. On the right we show the graph associated for a time-discretised approximation of Equation 1, where we've shown the incoming and outgoing connections of a single node (and in light grey the connections that directly go from the input to the output, represented by $\mathbf{W_{so}}$). Time goes from left to right. Note that, when the directions of the connections are turned around we again end up with a discrete-time convolution.

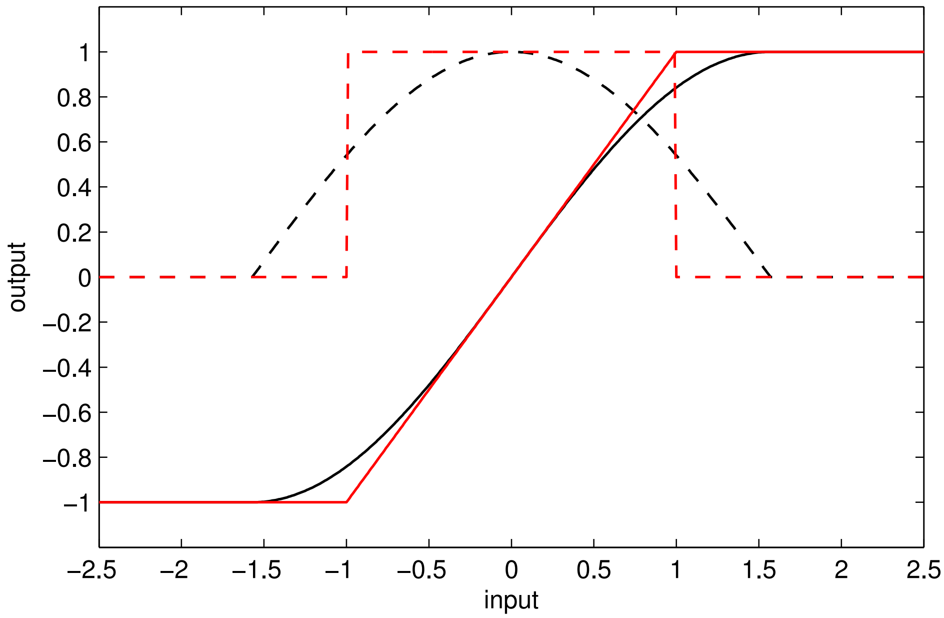

Supplementary Figure 2: Depiction of the true transfer function $\mathbf{g}(x)$ (black line) and its derivative (black dashed line), as used in one of the experiments versus their approximations in red. During simulation we use the true transfer function (black), but during the backpropagation we modulate with the Jacobian of the approximation (the binary function).

	Train FER	Test FER
Idealised training, $\mathbf{f}(x)$, all parameters	23.7%	29.8%
Idealised training, $\mathbf{f}(x)$, output masks only	35.5%	36.6%
Idealised training, $\mathbf{f}(x)$, internal parameters only	30.6%	33.3%
Non-ideal, $\mathbf{f}(x)$, $\epsilon = 0.1$	24.2%	29.9%
Non-ideal, $\mathbf{f}(x)$, $\epsilon = 0.2$	25.3%	30.5%
Non-ideal, $\mathbf{f}(x)$, $\epsilon = 0.5$	27.5%	31.8%
Idealised training, $\mathbf{g}(x)$	23.5%	29.5%
Non-ideal, $\mathbf{g}(x)$, binary Jacobian, $\epsilon = 0.1$	24.5%	30.3%

Supplementary Table 1: Frame error rates for different experiments

Supplementary Note 1

We will consider systems that are described by the following set of equations:

$$\begin{aligned}\mathbf{a}(t) &= \mathbf{f}\left(\left[\mathbf{W_{sa}} * \mathbf{s}\right](t) + \left[\mathbf{W_{aa}} * \mathbf{a}\right](t)\right) \\ \mathbf{o}(t) &= \left[\mathbf{W_{so}} * \mathbf{s}\right](t) + \left[\mathbf{W_{ao}} * \mathbf{a}\right](t)\end{aligned} \tag{1}$$

Here, $\mathbf{s}(t)$ is the input signal, $\mathbf{a}(t)$ is the internal state of the system and $\mathbf{o}(t)$ is the output of the system, and we've used the following notation for the convolution operation:

$$\left[\mathbf{W} * \mathbf{x}\right](t) = \int_0^\infty dt'\ \mathbf{W}(t')\mathbf{x}(t - t'). \tag{2}$$

By specific choices of the input signal $\mathbf{s}(t)$ and the impulse matrices $\mathbf{W_{xy}}(t)$, a wide range of common forms of neural networks are described by this system. First of all, let's consider the case where $\mathbf{W_{aa}}(t) = \mathbf{0}$, $\mathbf{W_{so}}(t) = \mathbf{0}$, and $\mathbf{W_{sa}}(t) = \delta(t)\mathbf{W_s}$, $\mathbf{W_{ao}}(t) = \delta(t)\mathbf{W_a}$. The equation reduces to:

$$\mathbf{o}(t) = \mathbf{W_a}\mathbf{f}\left(\mathbf{W}_s\mathbf{s}(t)\right), \tag{3}$$

which is nothing more than the description of a neural network with one hidden layer. A deep neural network (a multilayered perceptron) with L layers can be constructed also. We choose $\mathbf{W_{so}}(t) = \mathbf{0}$, $\mathbf{W_{sa}}(t) = \delta(t)\mathbf{W_s}$, $\mathbf{W_{aa}}(t) = \delta(t)\mathbf{W_a}$, and $\mathbf{W_{ao}}(t) = \delta(t)\mathbf{W_a}$, with the following respective definitions:

$$\mathbf{W_s} = \begin{bmatrix} \mathbf{W}_0 \\ \mathbf{0} \\ \mathbf{0} \\ \vdots \\ \mathbf{0} \end{bmatrix}, \tag{4}$$

$$\mathbf{W_a} = \begin{bmatrix} \mathbf{0} & \mathbf{0} & \mathbf{0} & \cdots & \mathbf{0} & \mathbf{0} \\ \mathbf{W}_1 & \mathbf{0} & \mathbf{0} & \cdots & \mathbf{0} & \mathbf{0} \\ \mathbf{0} & \mathbf{W}_2 & \mathbf{0} & \cdots & \mathbf{0} & \mathbf{0} \\ \mathbf{0} & \mathbf{0} & \mathbf{W}_3 & \cdots & \mathbf{0} & \mathbf{0} \\ \vdots & \vdots & \vdots & & \vdots & \vdots \\ \mathbf{0} & \mathbf{0} & \mathbf{0} & \cdots & \mathbf{W}_{L-1} & \mathbf{0} \end{bmatrix}, \tag{5}$$

$$\mathbf{W_a} = \begin{bmatrix} \mathbf{0} & \mathbf{0} & \mathbf{0} & \cdots & \mathbf{0} & \mathbf{W}_L \end{bmatrix} \tag{6}$$

If we further assume that $\mathbf{f}$ acts as a scalar function on each of the elements of its argument, we can rewrite the total system as:

$$\mathbf{o}(t) = \mathbf{W}_L\mathbf{f}(\mathbf{W}_{L-1}\mathbf{f}(\mathbf{W}_{L-2}\mathbf{f}(\mathbf{W}_{L-3}\mathbf{f}(\cdots\mathbf{W}_2\mathbf{f}(\mathbf{W}_1\mathbf{f}(\mathbf{W}_0\mathbf{s}(t))))))), \tag{7}$$

which is exactly the description of a deep neural network. Note that we do not need the transition matrices to be instantaneous (defined by delta functions) per se. If we hold $\mathbf{s}(t)$ fixed, the system may have internal delays, and we would simply need to hold the input for a long enough time for all internal delays to be resolved.

The system can quite simply be transformed into a recurrent neural network. We define the input signal to be piecewise constant with period Δ, and during each period the input is the next instance from a time series $\mathbf{s}_i$. We then simply need to define $\mathbf{W_{so}}(t) = \mathbf{0}$, $\mathbf{W_{sa}}(t) = \delta(t)\mathbf{W_s}$, $\mathbf{W_{aa}}(t) = \delta(t - \Delta)\mathbf{W_a}$, and $\mathbf{W_{ao}}(t) = \delta(t)\mathbf{W_o}$. This system will now act as a recurrent neural network, where each period Δ the system undergoes a state update.

$$\mathbf{o}(t) = \mathbf{W_o}\mathbf{a}(t), \tag{8}$$

$$\mathbf{a}(t) = \mathbf{f}(\mathbf{W_s}\mathbf{s}(t) + \mathbf{W_a}\mathbf{a}(t - \Delta)). \tag{9}$$

As may be inferred from these three simple examples, far more intricate or unusual neural architectures also fit in the framework described by equation 1. For instance, if we simply keep the input signal constant, i.e., $\mathbf{s}(t) = \mathbf{s}$ and we assume that under this condition, the system will eventually settle into a steady state, the convolution operations simply become matrix multiplications, and the internal state of the system is described by the following implicit equation:

$$\mathbf{a} = \mathbf{f}(\mathbf{W_{sa}}\mathbf{s} + \mathbf{W_{aa}}\mathbf{a}), \tag{10}$$

which almost never occurs in the neural networks literature, yet poses no problem on our physical setup, and such a system can be trained in the same manner as any system described by equation 1.

Supplementary Note 2

There are several ways to derive the gradients using the error backpropagation mechanism. While it is formally possible to start from equation 1 , it is conceptually easier to use a discrete-time approximation of the convolutions, and consider equation 1 to be a highly specific case of a neural network. First we will give a very brief explanation of the backpropagation algorithm for an arbitrary neural architecture, and next we consider the special case of the convolutional system used in this paper. For an in-depth proof and explanation of the equations we describe below, we refer to, e.g., [2]

All neural network architectures can be expressed by a directed graph, where each node performs a nonlinear function, and each edge is a weighted connection. When several edges arrive at a node, their values are added up before they enter the node nonlinearity. The error backpropagation algorithm works by first computing the gradient of the output w.r.t, a certain cost function, (which could be called the output error), and next propagating this error backwards through the directed graph by flipping the direction of each edge. Each edge is still a weighted connection, but during the error backpropagation phase, the error at each node is multiplied with the derivative of the nonlinear function (expressing the current sensitivity of the node). Once the error backpropagation phase is completed, each node in the network will be associated with an error value (the gradient of its input argument w.r.t. the cost function). The gradient of each weighted connection (edge) now is simply given by the error at the end times the activation (the output value of the neuron) at the front. This principle holds true for any kind of directed graph.

Instead of considering each node in the graph as a single, scalar function, one can easily expand this to multivariate systems. Now, each node represents a high-dimensional state and optionally a nonlinear function, and each edge represents a linear transformation matrix. Backwards propagation over such an edge is simply a multiplication with the transposed of this linear transformation matrix. Note that here already we can see the connection to reciprocity in physical systems. Instead of multiplication with the derivative, one multiplies with the jacobian. The gradient w.r.t. the connection matrix is now defined as the outer product of the error vector at the end of the directed edge with the activations at the start.

Suppose we have an input vector $\mathbf{s}$. We can then formally define any neural architecture as follows:

$$\mathbf{x}_i = \mathbf{W}_i^{\mathbf{s}}\mathbf{s} + \sum_k \mathbf{W}_{ik}\mathbf{a}_k \tag{11}$$

$$\mathbf{a}_i = \mathbf{f}_i\left(\mathbf{x}_i\right) \tag{12}$$

$$\mathbf{o} = \sum_k \mathbf{W}_k^{\mathbf{o}}\mathbf{a}_k + \mathbf{W}^{\mathbf{os}}\mathbf{s}, \tag{13}$$

where $\mathbf{a}_k$ represents the state of the k-th node of the neural graph, and $\mathbf{o}$ is the output on which we define a cost function. We assume that a subset of the transition matrices are equal to zero, such that no loops occur in the computational graph. Note that the transition matrices in these equation represent the *incoming* connections of each particular node in the graph. We define an error $\mathbf{e_o}$ as the gradient of the cost function C w.r.t. $\mathbf{o}$:

$$\mathbf{e_o} = \left(\frac{\partial C}{\partial \mathbf{o}}\right)^{\mathsf{T}}, \tag{14}$$

where we use the transpose to ensure $\mathbf{e_o}$ is a column vector (of the same size as $\mathbf{o}$). We can now define errors for each node in the graph as follows:

$$\mathbf{e}_i = \mathbf{J}_i^{\mathsf{T}}\left(\sum_k \mathbf{W}_{ki}^{\mathsf{T}}\mathbf{e}_k + \mathbf{W}_i^{\mathbf{o}\mathsf{T}}\mathbf{e_o}\right), \tag{15}$$

with

$$\mathbf{J}_i = \frac{\partial \mathbf{f}_i(\mathbf{x}_i)}{\partial \mathbf{x}_i}. \tag{16}$$

Here, all the transition matrices in these equations represent the *outgoing* connections of each node in the graph, which represents the fact the error propagates *backwards* through the graph. Note that the mathematical interpretation of each error $\mathbf{e}_i$ corresponds to the total derivative of C w.r.t. the input argument $\mathbf{x}_i$ as put forward in equation 12:

$$\mathbf{e}_i = \left(\frac{dC}{d\mathbf{x}_i}\right)^{\mathsf{T}}. \tag{17}$$

Finally, gradients w.r.t. each transition matrix in the network can be defined as follows:

$$\frac{dC}{d\mathbf{W}_{ij}} = \mathbf{e}_j \mathbf{a}_i^{\mathsf{T}}, \quad \frac{dC}{d\mathbf{W}_i^{\mathbf{s}}} = \mathbf{e}_i \mathbf{s}^{\mathsf{T}}, \quad \frac{dC}{d\mathbf{W}_i^{\mathbf{o}}} = \bar{\mathbf{e}} \mathbf{a}_i^{\mathsf{T}}, \quad \frac{dC}{d\mathbf{W}^{\mathbf{os}}} = \bar{\mathbf{e}} \mathbf{s}^{\mathsf{T}}, \tag{18}$$

which are defined such that they are of the same size as the matrices themselves.
Note that these equations hold true for any neural architecture. If some of the transition matrices are always equal (which is the case for the convolution we will use), one simply needs to add up the gradients that result from all the single outer products described above.
When making a discrete-time approximation of the convolutions given in equation 1, it can be reduced to a directed graph with the properties described above. First of all, we use the following approximation for the convolution:

$$[\mathbf{W} * \mathbf{y}](t) \approx \delta_t \sum_k \mathbf{W}(k\delta_t)\mathbf{y}(t - k\delta_t), \tag{19}$$

which converges to the proper convolution for $\delta_t \to 0$. We have represented the associated computational graph in the bottom of Supplementary Figure 1, where we've depicted the incoming and outgoing connections of a single node (time instance) of the state vector $\mathbf{a}(t)$. Note that the connections are shared over time, i.e., the incoming and outgoing connection matrices for different time instance of $\mathbf{a}(t)$ are the same. Also the incoming and outgoing connection matrices that connect the nodes representing $\mathbf{a}(t)$ are symmetric, in the sense that the transition matrix connecting the node representing $a(t-d)$ to $a(t)$ is always equal to the transition matrix connecting node $a(t)$ to $a(t+d)$.
We will use the graph representation of the time-discretised approximation of Equation 1. We start by defining $\mathbf{e_o}(t)$ as the derivative of the cost function w.r.t. $\mathbf{o}(t)$, We time discretise $\mathbf{e_o}(t)$, and write down the time-discretised version of Equation 1:

$$\begin{aligned}
\mathbf{a}(i\delta_t) &= \mathbf{f}\left(\delta_t \sum_k \mathbf{W_{sa}}(k\delta_t)\mathbf{s}((i-k)\delta_t) + \delta_t \sum_k \mathbf{W_{aa}}(k\delta_t)\mathbf{a}((i-k)\delta_t)\right) \\
\mathbf{o}(i\delta_t) &= \delta_t \sum_k \mathbf{W_{so}}(k\delta_t)\mathbf{s}((i-k)\delta_t) + \delta_t \sum_k \mathbf{W_{ao}}(k\delta_t)\mathbf{a}((i-k)\delta_t)
\end{aligned} \tag{20}$$

We can now simply apply equation 15 for backpropagation through a neural architecture. By switching the outgoing with the incoming connections we can write down:

$$\mathbf{e_a}(i\delta_t) = \mathbf{J}^{\mathsf{T}}(i\delta_t)\left(\delta_t \sum_k \mathbf{W}_{\mathbf{ao}}^{\mathsf{T}}(k\delta_t)\mathbf{e_o}((i+k)\delta_t) + \delta_t \sum_k \mathbf{W}_{\mathbf{aa}}^{\mathsf{T}}(k\delta_t)\mathbf{e_a}((i+k)\delta_t)\right) \tag{21}$$

When $\delta_t \to 0$, this reduces again to the continuous-time equation for the backpropagation as provided in the main text. When $\mathbf{s}(t)$ is a trainable representation of another kind of signal, we will also need to propagate the error to the input signal $\mathbf{s}(t)$, which is given by:

$$\mathbf{e_s}(i\delta_t) = \delta_t \sum_k \mathbf{W}_{\mathbf{sa}}^{\mathsf{T}}(k\delta_t)\mathbf{e_a}((i+k)\delta_t) + \delta_t \sum_k \mathbf{W}_{\mathbf{so}}^{\mathsf{T}}(k\delta_t)\mathbf{e_o}((i+k)\delta_t) \tag{22}$$

This equation too, reduces to the equation given in the main article when $\delta_t \to 0$. If errors are computed over a time span $t \in \{0 \cdots T\}$, we can define gradients as

$$\frac{dC}{d\mathbf{W_{sa}}(t)} = \int_0^{T-t} dt' \mathbf{e_a}(t'+t)\mathbf{s}^\mathsf{T}(t') \tag{23}$$

$$\frac{dC}{d\mathbf{W_{aa}}(t)} = \int_0^{T-t} dt' \mathbf{e_a}(t'+t)\mathbf{a}^\mathsf{T}(t') \tag{24}$$

$$\frac{dC}{d\mathbf{W_{so}}(t)} = \int_0^{T-t} dt' \mathbf{e_o}(t'+t)\mathbf{s}^\mathsf{T}(t') \tag{25}$$

$$\frac{dC}{d\mathbf{W_{ao}}(t)} = \int_0^{T-t} dt' \mathbf{e_o}(t'+t)\mathbf{a}^\mathsf{T}(t'). \tag{26}$$

Here the integral stems from the fact that we need to sum op all contributions to the gradients, each from different time steps. This summation becomes an integral in continuous time. Note that de mathematical interpretations of $\mathbf{e_o}(t)$, and $\mathbf{e_s}(t)$ are similar to that in Equation 17, i.e., they are the total derivatives of the cost function w.r.t. the output signal $\mathbf{o}(t)$ and the input signal $\mathbf{s}(t)$. For $\mathbf{e_a}(t)$, it is the total derivative w.r.t. the input argument of the nonlinearity. The interpretation is that these signals represent how the respective signals should be adapted at any point in time, *accounting for all future changes in cost this adaptation would have.* This information – how changes to the internal variables would manifest themselves for the cost function over time – is what is accumulated during the backpropagation process.

Supplementary Note 3

If we use a physical dynamic system, the input signal we send in is a continuous-time signal. When we wish to process a discrete time series, which is quite typical for many applications, we need to convert the discrete time signal to a continuous time signal. We will do this using the so-called masking principle, first described in [1]. Here we will use a slightly generalised definition. First of all we define an encoding which transforms the vector $\mathbf{x}_i$, the i-th instance of a time series, into a continuous time signal segment $\mathbf{s}_i(t)$:

$$\mathbf{s}_i(t) = \mathbf{s}_b(t) + \mathbf{M}(t)\mathbf{x}_i \quad \text{for} \quad t \in [0 \cdots P]\,, \tag{27}$$

where P is the masking period and $\mathbf{s}_b(t)$ is a bias time trace. The matrix $\mathbf{M}(t)$ are the so-called input masks, defined for a finite time interval of duration P. The input signal $\mathbf{s}(t)$ is now simply the time-concatenation of the finite time segments $\mathbf{s}_i(t)$

The output encoding works in a very similar fashion. If there is a time series $\mathbf{y}_i$ which represents the output associated with the i-th instance of the input time series, we can define an output mask $\mathbf{U}(t)$. We divide the time trace of the system output $\mathbf{o}(t)$ into segments $\mathbf{o}_i(t)$ of duration P. The i-th network output instance is then defined as

$$\mathbf{y}_i = \mathbf{y}_b + \int_0^P dt\, \mathbf{U}(t)\mathbf{o}_i(t), \tag{28}$$

with $\mathbf{y}_b$ a bias vector. The process described here is essentially a form of time multiplexing. The backpropagation phase happens very similarly. Suppose we have a time series with instances $\mathbf{e}_i$, which are the gradient of a cost function w.r.t. the output instances $\mathbf{y}_i$. Completely equivalent to the input masking we can now define the error signal $\mathbf{e_o}(t)$ as a time concatenation of finite time segments $\mathbf{e}^i_{\mathbf{o}}(t)$:

$$\mathbf{e}^i_{\mathbf{o}}(t) = \mathbf{U}^{\mathsf{T}}(t)\mathbf{e}_i. \tag{29}$$

Using this signal as input to the system during backpropagation will provide us with an error $\mathbf{e_s}(t)$. Exactly equivalent to the output signal, we will now partition $\mathbf{e_s}(t)$ as a time-concatenation of finite segments $\mathbf{e}^i_{\mathbf{s}}(t)$. Finally, the gradient w.r.t. the input mask $\mathbf{M}(t)$ is given by:

$$\frac{dC}{d\mathbf{M}(t)} = \sum_i \mathbf{e}^i_{\mathbf{s}}(t)\mathbf{x}_i^{\mathsf{T}}. \tag{30}$$

Note that in reality, the signals will be sampled using measurement equipment, and we will define $\mathbf{M}(t)$ and $\mathbf{U}(t)$ as discrete-time signals.

Supplementary Note 4

Here we present the supplementary material on the simulations performed with the conceptual electro-optical setup. First we will present the different approximations of the physical system, next we will provide the results for all these experiments, and finally we will argue what the potential obtainable speed of such a system can be. The electro-optical setup can be modeled by the following equation:

$$\mathbf{a}(t) = \mathbf{f}\left(\mathbf{W}\mathbf{a}(t-D) + \mathbf{s}(t)\right), \tag{31}$$

where

$$f(x) = \begin{cases} -1 & \text{if } x \leq -1 \\ x & \text{if } -1 < x < 1 \\ 1 & \text{if } x \geq 1. \end{cases} \tag{32}$$

We will now consider different levels of non ideal behaviour.

- In the ideal scenario, backpropagation through time should be implemented by the following equation

  $$\mathbf{e_a}(t) = \mathbf{J}(t)\left(\mathbf{W}^\mathsf{T}\mathbf{e_a}(t+D) + \mathbf{e_o}(t)\right). \tag{33}$$

  We will perform simulations for this idealised scenario to have a baseline performance to compare with when we study different degrees of non-ideal behaviour.

- As a second baseline, we will consider what happens when we only train the parameters influencing the dynamics (the input masks and the mixing matrix $\mathbf{W}$) under constant and randomly drawn output masks, or only the output weights (with only scaling of input masks and the mixing matrix $\mathbf{W}$ optimised). This will be an indicator of how much can be gained by training each group of parameters separately.

- The transfer function of the electro-optic modulator will still be present during the backpropagation phase, as we can not modulate the intensity to levels lower than zero or higher than the laser source intensity. Therefore, we will run simulations in which we truncate the error signal before feeding it back into the system. Essentially this means the backpropagation will run according to the following equation:

  $$\mathbf{e_a}(t) = \mathbf{J}(t)\left(\mathbf{W}^\mathsf{T}\mathbf{f}(\mathbf{e_a}(t+D)) + \mathbf{e_o}(t)\right). \tag{34}$$

  Note that, since scaling of the error signal $\mathbf{e_o}(t)$ is arbitrary, such that we could simply scale down $\mathbf{e_o}(t)$ sufficiently such that the backpropagated error $\mathbf{e_a}(t)$ never reach the truncation levels. Realistically, however, there will be a certain amount of noise, and downscaling $\mathbf{e_o}(t)$ will cause it to fall under the noise threshold, making it useless. Therefore, in any realistic scenario we will need to strike a balance between the effects of the nonlinearity and the effects of noise.
  We performed three different experiments where we test different levels of noise superimposed on both the signal in the forward pass and the backward pass. The equations are described by

  $$\mathbf{a}(t) = \mathbf{f}\left(\mathbf{W}\mathbf{a}(t-D) + \mathbf{s}(t) + \epsilon\mathbf{n}(t)\right), \tag{35}$$

  $$\mathbf{e_a}(t) = \mathbf{J}(t)\left(\mathbf{W}^\mathsf{T}\mathbf{f}(\mathbf{e_a}(t+D)) + \mathbf{e_o}(t) + \epsilon\mathbf{n}(t)\right), \tag{36}$$

  where $\mathbf{n}(t)$ is i.i.d. Gaussian noise sampled for each "measurement" (time step) in the simulation from a standard normal distribution, and ϵ is a scaling factor. Note that we ensured that noise is present "in the loop". We consider three values for ϵ: 0.1, 0.2 and 0.5, corresponding to a signal-to-noise ratio of roughly 18 dB, 12 dB and 3dB in the forward pass, respectively (the input signal had a standard deviation of about 0.75). During backpropagation we normalised $\mathbf{e_o}(t)$ by dividing

it with its standard deviation, and next scaled it with a factor 0.2 for $\epsilon = 0.1$ and $\epsilon = 0.2$, and a factor 0.5 for $\epsilon = 0.5$ (empirically found to work well). Note that this implies that the power of the input signal is roughly that of the noise present within the system.

- Thirdly, we will consider the case of deviations in the nonlinear behaviour. The transfer function we consider here is piecewise linear. Realistically, however, there could be deviations from such a mathematically ideal behaviour. For instance their could be saturation effects near the truncation thresholds, or other non-ideal effects. Note that in principle we could measure such effects, and try to keep them into account during the backprop phase. In this particular case, however it would mean we lose the advantage of the simplicity of the binary derivative.
  Multiple devices have been demonstrated that have a linear modulation response [3, 4, 5, 6]. Here, however we will consider what happens if we use modulation using a sine transfer function (which is the case in Mach-Zehnder interferometers, the most widely used commercially available modulator). We will use the following transfer function instead of $\mathbf{f}(x)$:

$$\mathbf{g}(x) = \begin{cases} -1 & \text{if } x \leq -\pi/2 \\ \sin(x) & \text{if } -\pi/2 < x < \pi/2 \\ 1 & \text{if } x \geq \pi/2. \end{cases} \tag{37}$$

  What we wish to find out in this experiment is the effect of an incorrect Jacobian. Suppose for instance that continuous modulation with the correct Jacobian is difficult to achieve in hardware, one might resort to an approximation. Here, in order to compute the Jacobian, we will approximate this nonlinearity with $\mathbf{f}(x)$, the piecewise linear function used before. This means that during the backpropagation phase we modulate the feedback signal with the approximate (binary) Jacobian. Note that this happens on top of all other non-idealities such as noise and nonlinearity in the loop during backpropagation. Supplementary Figure 2 shows a comparison of $\mathbf{f}(x)$, $\mathbf{g}(x)$ and their respective derivatives. For this experiment we also include noise at a level of $\epsilon = 0.1$. As a sanity check, we also run an experiment in which we measure performance for the transfer function $\mathbf{g}(x)$ in the ideal scenario (i.e. no noise and no nonlinearity in the backpropagation)

Note that in all experiments we included the same level of noise during testing as we used during training.

The resulting train and test frame error rates (FER) are listed in Supplementary Table 1. The baseline train error rate for the ideal scenario is 29.8%. From the results in which only the output masks are trained or only the output weights, it is apparent that most can be gained from optimising the internal parameters. This is important as those parameters are the ones that are affected by non-ideal behavior in the backpropagation phase (output masks do not need the backpropagation phase to be trained, but are rather trained from forward measurements only, as is the case in the reservoir computing setup).
When considering the results for non-ideal behaviour, we see that the FER is not that strongly affected by increasing levels of noise. Even for $\epsilon = 0.5$, where noise intensity is nearly that of the signal itself, the test FER only raises with 2% compared to the ideal scenario. The increase is somewhat more apparent in the training error rate, where it raises with almost 4%.
The sine-transfer function $\mathbf{g}(x)$ instead of the piecewise-constant $\mathbf{f}(x)$ has in the ideal scenario a slightly better train and error rate. When the Jacobian of $\mathbf{g}(x)$ is approximated with a binary function, FER increases with roughly one percent for the train and test data, bearing witness to a remarkable robustness against systematic deviations during the backpropagation phase.

Supplementary Discussion

Here we will try to estimate the operational speed that is achievable for the opto-electronic setup. As all components of the network itself are passive optical fibers, speed will essentially be limited by the bandwidth of the opto-electronic "neurons".

Signal generation and measurement can happen at several Gigasamples per second (high-end arbitrary waveform generators and oscilloscopes can even reach sample rates of up to 50 Gigasamples per second). Optical modulators, including those with a linear response function, commonly have bandwidths well into several GHz. The speed of the design will therefore be mostly influenced by the speed of the photodiode that measures the light intensity, and by the system used to modulate the Jacobian.

The measurement speed of a photodiode is commonly limited by the bandwidth of the required amplification. High-end photodiodes can have bandwidths reaching up to 100GHz (see for instance `http://www.hhi.fraunhofer.de/en/fields-of-competence/photonic-components/topics/detectors.html`). Modulation of the Jacobian using an electronic analogue switch is unfortunately relatively slow compared to the previously mentioned devices. Typically, switching times are several nanoseconds long, reducing the obtainable sample rate to below 1 GHz. One could, however, modulate with the Jacobian in the optical domain using an additional optical switch. Especially in the case of a binary Jacobian, one could use a fast 2X2 Mach-Zehnder-based switch that receives at its input both the transmitted signal and a constant signal representing zero. Modulating with the Jacobian then simply requires switching the output between these two input signals. As a directional coupler has the same operating principle as a normal optical modulator, it can obtain similar speeds too, well into several Gigahertz.

Considering the bandwidths of these devices, the opto-electronic setup as described here can optimistically reach sample rates in the order of a Gigasample per second. It is hard to foresee, however, what levels of noise are to be expected (typically measurements get noisier at higher sample rates). Still as we have demonstrated, noise levels need to be quite significant before performance is notably affected.

Note that, at 1 Gigasample per second, all measurements we did for training the system could be performed in 50 seconds (50 samples per frame, 50 frames per training batch, 200 batches per training iteration, and 50,000 training iterations, double for both forward and backwards direction). This would not include the time required for computing gradients, updating parameters, and other forms of experimental overhead, but it exemplifies the speed advantages a physical analog network can potentially offer. In contrast, training the network in simulation takes between 24 and 30 hours, of which about 95% goes to simulation of the network in the forward and backwards direction. This means that the electro-optical concept would still be highly advantageous even when the true obtainable speed is much lower (e.g., 10 to 100 Megasamples per second, which corresponds to about two hours to ten minutes).

Supplementary References

[1] Lennert Appeltant, Miguel Cornelles Soriano, Guy Van der Sande, Jan Danckaert, Serge Massar, Joni Dambre, Benjamin Schrauwen, Claudio R Mirasso, and Ingo Fischer. Information processing using a single dynamical node as complex system. *Nature communications*, 2:468, 2011.

[2] Christopher M. Bishop. *Pattern Recognition and Machine Learning (Information Science and Statistics)*. Springer, August 2006.

[3] Akimasa Kaneko, Hiroshi Yamazaki, and Yutaka Miyamoto. Linear optical modulator. In *Optical Fiber Communication Conference*, pages W3K–5. Optical Society of America, 2014.

[4] Xiaobo Xie, Jacob Khurgin, Jin Kang, and F-S Chow. Linearized mach-zehnder intensity modulator. *Photonics Technology Letters, IEEE*, 15(4):531–533, 2003.

[5] Hiroshi Yamazaki, Hiroshi Takahashi, Takashi Goh, Yasuaki Hashizume, Shinji Mino, and Yutaka Miyamoto. Linear optical iq modulator for high-order multilevel coherent transmission. In *Optical Fiber Communication Conference*, pages OM3C–1. Optical Society of America, 2013.

[6] Peng Yue, Xiang Yi, Qian-Nan Li, Tuo Wang, and Zeng-Ji Liu. Mmi-based ultra linear electro-optic modulator with high output rf gain. *Optik-International Journal for Light and Electron Optics*, 124(17):2623–2626, 2013.
